# Production of recombinant human xCT (SLC7A11) and reconstitution in proteoliposomes for functional studies

**DOI:** 10.3389/fphys.2022.993626

**Published:** 2022-09-06

**Authors:** Michele Galluccio, Mariafrancesca Scalise, Gilda Pappacoda, Martina Scarpelli, Marcella Bonanomi, Daniela Gaglio, Cesare Indiveri

**Affiliations:** ^1^ Unit of Biochemistry and Molecular Biotechnology, Department DiBEST (Biologia Ecologia Scienze della Terra), University of Calabria, Arcavacata di Rende, Italy; ^2^ Department of Biotechnology and Biosciences, University of Milano-Bicocca, Milan, Italy; ^3^ ISBE.IT/Centre of Systems Biology, Milan, Italy; ^4^ Institute of Molecular Bioimaging and Physiology (IBFM), National Research Council (CNR), Milan, Italy; ^5^ CNR Institute of Biomembranes, Bioenergetics, and Molecular Biotechnologies (IBIOM), Bari, Italy

**Keywords:** xCT, SLC7A11, liposomes, over-expression, cystine, redox control, reactive oxygen species, glutamate

## Abstract

The plasma membrane transporter xCT belongs to the SLC7 family and has the physiological role of mediating the exchange of glutamate and cystine across the cell plasma membrane, being crucial for redox control. The xCT protein forms a heterodimer with the ancillary protein CD98. Over the years, xCT became a hot pharmacological target due to the documented over-expression in virtually all human cancers, which rely on cystine availability for their progression. Notwithstanding, several unknown aspects of xCT biology still exist that require a suitable single protein experimental model, to be addressed. To this aim, the recombinant host *Escherichia coli* has been exploited to over-express the human isoform of xCT. In this widely used and low-cost system, the optimization for growth and protein production has been achieved by acting on the metabolic needs of the bacterial strains. Then, the His-tagged protein has been purified by Ni^2+^-chelating chromatography and reconstituted in proteoliposomes for transport activity assays. The expressed protein was in a folded/active state allowing functional and kinetic characterization. Interestingly, the features of the recombinant protein meet those of the native one extracted from intact cells, further confirming the suitability of *E. coli* as a host for the expression of human proteins. This study opens perspectives for elucidating other molecular aspects of xCT, as well as for studying the interaction with endogenous and exogenous compounds, relevant to human health.

## Introduction

SLC7A11, also known as xCT, has been described as a sodium independent obligatory exchanger of cystine and glutamate localized at the cell membrane (Sato et al., 1999). As in the case of other members of the SLC7 family, the xCT transporter forms a heterodimer with the second member of the SLC3 family, CD98 (SLC3A2) also named 4F2hc (Jyotsana et al., 2022). The xCT/CD98 heterodimer is historically known as system xc^−^, with xCT being the un-glycosylated light chain subunit and CD98 being the type II glycoprotein heavy chain. The interaction between the two proteins occurs at the level of two conserved cysteine residues, which form a disulfide bridge. xCT appears in many papers mostly describing the involvement of this transporter in various human cancers (Chio and Tuveson, 2017; Ji et al., 2018; Shin et al., 2018; Combs and DeNicola, 2019; Lin et al., 2020; Koppula et al., 2021), especially in those that are resistant to chemotherapy and radiotherapy (Lin et al., 2020). Over the years, this phenomenon has been linked to the role played by xCT in cancer biology (Kinoshita et al., 2013; Liu et al., 2020; Koppula et al., 2021). Since this transporter is responsible for the uptake of cystine, it provides cells with about half of the cell cystine pool (Ghasemitarei et al., 2019), required for regulation of the cell redox state; once in cells, cystine is rapidly converted to cysteine through a NADPH dependent reaction in the cytosolic space (Go and Jones, 2008). Then, cysteine, through a two-step process catalyzed by *γ*-glutamylcysteine synthetase and glutathione synthetase, is converted to glutathione, a key player of the redox state of cells (Lu, 2009). Furthermore, cysteine is also the precursor of taurine and hydrogen sulfide, which are involved in redox signalling (Stipanuk et al., 2006; Jong et al., 2012; Combs and DeNicola, 2019). Even if cysteine can derive from protein degradation and *de novo* synthesis, cancer cells dramatically increase their demand for this amino acid to cope with the modified metabolic requirement and to maintain the redox homeostasis, as it occurs in the case of glutamine (Chio and Tuveson, 2017; Ji et al., 2018; Shin et al., 2018; Combs and DeNicola, 2019; Lin et al., 2020; Koppula et al., 2021). In the last years, xCT over-expression in human cancer has been linked with ferroptosis, an iron-dependent cell death characterized by excessive accumulation of peroxidated polyunsaturated fatty acids (PUFAs) (Koppula et al., 2018). Indeed, the increased levels of cystine uptake and, in turn, cysteine and glutathione levels, slow down ferroptosis promoting cancer cell survival. Besides the role in regulating ferroptosis, xCT affects tumor microenvironment by exporting glutamate that in cancer cells acts as an oncogenic signalling molecule favouring tumor proliferation, invasion, and metastasis (Lin et al., 2020). Moreover, xCT modulates the degeneration of motor neurons in mice affected by amyotrophic lateral sclerosis; based on this observation, the inhibition of xCT activity could slow down the disease progression (Mesci et al., 2015). Interestingly, the expression of this transporter increases also in multiple sclerosis, providing a link between inflammation and excitotoxicity in demyelinating diseases (Pampliega et al., 2011). Due to the above-described involvement in human pathologies, xCT is nowadays considered an attractive target for anticancer therapies, since inhibition of xCT function will increase ferroptosis triggering cancer cell death (Sato et al., 2018). In line with this scenario, many efforts have been dedicated to obtain the 3D structure of the xCT protein. The first structure of the heterodimer has been solved by Cryo-EM, after expression in HEK293S GnTI cells, employing a construct subjected to consensus mutagenesis reaching more than 35% sequence mutation (Oda et al., 2020). More recently, the structure of xCT/CD98 heterodimer has been obtained by Cryo-EM in both the apo (PDB:7P9U) and glutamate bound (PDB:7P9V) states (Parker et al., 2021). The same experimental approach has been used by another group to obtain the structure of xCT/CD98 bound to erastin (PDB 7EPZ), a well-known ferroptosis inducer, shedding light on the molecular basis of ferroptosis (Yan et al., 2022). Notwithstanding the huge efforts in deepening the knowledge of xCT structure, several functional and kinetic aspects of the transporter are still obscure and need to be addressed. As for other membrane transporters, the main reason for such a delay is the intrinsic difficulty in handling these proteins with a unique methodological approach. Indeed, the intact cell system approach, mainly used for measuring the transport activity of xCT, is affected by interferences due to the presence of other molecular systems with similar or overlapping substrate specificity. This feature hampers the accurateness of side-specific functional and kinetic investigations that become mandatory for the complete characterization of the system, especially for an antiporter as in the case of xCT. To this aim, we here report the successful over-expression and purification of the recombinant human xCT in *E. coli*, and its reconstitution in proteoliposomes in an active form. This exquisitely *in vitro* model constitutes the experimental bases for improving structure/function relationships studies together with site-directed mutagenesis approaches for designing and testing potential drugs and effectors.

## Materials and methods

Chemicals used for experiments, protease inhibitor cocktail (P8849), Nickel Affinity Gel (HIS-Select^®^—P6611) and the Monoclonal Anti-polyHistidine-Peroxidase antibody (A7058), Amberlite XAD-4, egg yolk phospholipids, Sephadex G-75, L-glutamate from Merck Life Science; restriction endonucleases and specific reagents for cloning from Thermo scientific; *E. coli* Rosetta (DE3) strain from Novagen; MegaMan Human Transcriptome Library, BL21 codon plus RIL from Agilent technologies. L-[^3^H]-glutamate from Perkin Elmer, C_12_E_8_ from TCI Europe, bacterial lipids from Avanti polar Lipids.

### Cloning of human xCT

The cDNA encoding for human xCT transporter (SLC7A11) (UniProtKB: Q9UPY5 ***;*** GenPept accession no. NP_055146.1), was amplified from MegaMan Human Transcriptome Library using the following specific primers: 5′- CCGGAA​TTCTAT​GGT​CAG​AAA​GCC​TGT​TGT​GTC​CA-3' (forward) and 5′- ACG​CGTC​GACTCA​TAA​CTT​ATC​TTC​TTC​TGG​TAC​A-3' (reverse), respectively. The amplified sequence was cloned between *Eco*RI and *Sal*I restriction sites of the pH6EX3 expression vector. The resulting recombinant plasmid, defined as pH6EX3-hxCT, encodes a 6His-tagged fusion protein corresponding to the hxCT carrying the extra N-terminal MSPIHHHHHHLVPRGSEASNS sequence.

### Expression of human xCT transporter

To produce the 6His-hxCT recombinant protein, *E. coli* BL21 codon plus or Rosetta (DE3) cells, were transformed with the pH6EX3-hxCT construct. The selection of transformed colonies was performed on LB-agar plates added with 100 μg/ml ampicillin and 34 μg/ml chloramphenicol. Different media (LB or Terrific) prepared in the absence or presence of different glucose concentrations were assayed. According to the number of conditions to be tested, colonies were inoculated in a different volume of a specific medium and cultured overnight at 37°C under rotary shaking (160 rpm). The day after, the cultures were diluted at 1:20 in fresh medium added with the specific antibiotics and specifically treated. In particular, when the optical density measured at 600 nm wavelength was about 0.8–1, the growth temperature was lowered to 28°C and different IPTG concentrations (from 0.05 to 0.4 mM) were tested to induce protein expression up to 8 h except for one aliquot, grown in absence of inducer, (negative control). Every 2 h, aliquots were collected and centrifuged at 3,000 × g, and 4°C for 10 min; the pellets were stored at −20°C. A bacterial pellet aliquot, after thawing, was dissolved in a resuspension buffer (20 mM Hepes/Tris, 300 mM NaCl pH 7.5) and added with protease inhibitor cocktail according to manufacturer instructions. The bacterial suspensions were sonified in an ice bath for 5 min (pulse of 1 s on, and 1 s off) at 30%, using a Branson SFX 550 sonifier. The insoluble cell fractions were analyzed by SDS-PAGE and Western blotting.

### Purification of the hxCT protein

To purify 6His-hxCT protein, the pellet of 24 ml bacterial suspension was washed twice with 0.1 M Tris/HCl pH 8.0 and centrifuged at 12,000 × g for 10 min. The new pellet was solubilized by adding 1.6 ml of 8 M urea, 80 μl of 500 mM DTE, 216 μl of 10% sarkosyl, 1 mM glutamate, and mixed for 30 min in a fixed angle rotator for tubes. Then, 1 ml of a renaturing buffer (A) containing: 0.1% sarkosyl, 200 mM NaCl, 10% glycerol, 1 mM glutamate, and 20 mM Tris/HCl at pH 8.0 was added and the rotation continued for 30 min in the same conditions. After this time, the sample was centrifuged (12,000 × g, 10 min, 4°C), and the supernatant was added to a His select Ni^2+^ affinity gel column (0.5 × 7.5 cm; 3 ml= 1 Column Volume) equilibrated with 10 column volumes of a renaturing buffer A, and then mixed for 1 h at 4°C in a fixed angle rotator for tubes. Then, the protein resin mix was transferred into a column and packed by gravity. Then, 5 ml of washing buffer (0.1% C_12_E_8_, 200 mM NaCl, 10% glycerol, 1 mM glutamate, 5 mM DTE, 20 mM Tris/HCl at pH 8.0) were added to remove unbound proteins. A second washing step was performed with 10 ml of the same buffer added with 10 mM imidazole. Then, 5 ml of washing buffer (0.3% C_12_E_8_, 200 mM NaCl, 10% glycerol, 1 mM glutamate, 5 mM DTE, 20 mM Tris/HCl at pH 8.0) added with 10 mM imidazole were added to increase detergent concentration required for protein solubility. The elution was performed using 8 ml of elution buffer composed of: 0.3% C_12_E_8_, 200 mM NaCl, 10% glycerol, 1 mM glutamate, 5 mM DTE, 20 mM Tris/HCl at pH 8.0, and 500 mM imidazole. Fractions of 1 ml were collected and analyzed by SDS-PAGE and western blotting. The purified xCT was present in fractions 4 and 5. The fractions were pulled together and passed through a PD-10 column for removing imidazole using a buffer composed of 0.3% C_12_E_8_, 200 mM NaCl, 10% glycerol, 5 mM DTE, 1 mM glutamate, 20 mM Tris/HCl at pH 8.0. The protein was then used for reconstitution in proteoliposomes as described in the following paragraph.

### Cell culture

A549 cells were maintained in Dulbecco’s Modified Eagle Medium (DMEM) supplemented with 10% (v/v) fetal bovine serum (FBS), 2 mM glutamine, and Pen/Strep as antibiotics. Cells were grown on 10 cm^2^ plates at 37°C in a humidified incubator and a 5% CO_2_ atmosphere. The xCT/CD98 complex was solubilized from A549 pellets with RIPA buffer supplemented with 1% C_12_E_8_ and protease inhibitor cocktail (from Merck Life Science). The solubilized suspension was incubated on ice for 30 min and then centrifuged (12,000 × g, 20 min, 4°C). After centrifugation, proteins in the supernatant were quantified using the Lowry Folin method and subjected to SDS-PAGE 12% for western blot analysis against xCT and CD98.

### Reconstitution of the hxCT transporter into proteoliposomes

The desalted protein was used for reconstitution in liposomes. The composition of the mixture was: 5 μg (even if no change of transport activity was observed in a range from 3 to 7 μg) of the purified protein in desalting buffer (or 150 μg of total lysate from A549 cell pellet), 100 μl of 10% C_12_E_8_, 100 μl of 10% egg yolk phospholipids in the form of sonicated liposomes prepared with 7.5% cholesterol as previously described (Cosco et al., 2020), 10 mM glutamate (or different concentrations as indicated in figure legends), 10 mM DTE and 20 mM HEPES Tris pH 7.5 in a final volume of 700 μl. The mixture was then incubated for 90 min under rotatory stirring (1,200 rpm) at 23°C with 0.5 g of Amberlite XAD-4, for detergent removal from mixed micelles. Upon detergent removal the final lipid concentration reached 10 mg/ml and the protein/lipid ratio was 1:2,000. All procedures were performed at room temperature.

### Transport measurements

To remove the external compounds, 600 μl of proteoliposomes was passed through a Sephadex G-75 column (0.7 cm diameter × 15 cm height) equilibrated with a buffer containing 20 mM Hepes Tris pH 7.5. The eluate was collected and divided into 100 μl samples used for transport assay. The uptake was started by adding 10 μM [^3^H]-glutamate (50.8 Ci/mmol) at room temperature and stopped with 250 μM HgCl_2_, an inhibitor of xCT, at the desired times, as indicated in the figure legends. In the control sample, the same inhibitor was added at time zero, according to the inhibitor stop method (Cosco et al., 2020). Then, to remove the external radioactivity, 100 μl of each sample were passed through a Sephadex G-75 column (0.6 diameter × 8 cm height), buffered with 50 mM NaCl. Liposomes were eluted with 1 ml of the same buffer and collected in a 3 ml of scintillation mixture, for radioactivity counting. The experimental values were analyzed by subtracting to each sample the respective blanks.

### Protein quantification

Protein concentration was measured by the method of Lowry, modified for the presence of detergents (Dulley and Grieve, 1975). Proteins were separated by SDS–PAGE on 12% polyacrylamide gels performed according to Laemmli (Laemmli, 1970), using the Hoefer SE260 mini-vertical unit and stained by Coomassie-brilliant blue or using a stain-free technology (Bio-Rad). Quantitative evaluation of Coomassie-stained or stain-free visualized protein bands was carried out using the Chemidoc imaging system equipped with Quantity One software (Bio-Rad).

### Western blotting

Recombinant human xCT protein was immuno-detected using the Monoclonal Anti-polyHistidine-Peroxidase antibody 1:10,000. The reaction was detected by Electro Chemi Luminescence (ECL). Native xCT and CD98 proteins were immunodetected using polyclonal anti xCT 1:1,000 and anti CD98 1:1,000 antibodies, respectively. The incubation with primary antibodies was conducted overnight at 4°C. The reaction was detected by Electro Chemi Luminescence (ECL) assay after 1 h incubation with secondary antibody anti-rabbit 1:5,000.

## Results and discussion

### Expression in *E. coli* and purification of the human xCT

**FIGURE 1:**
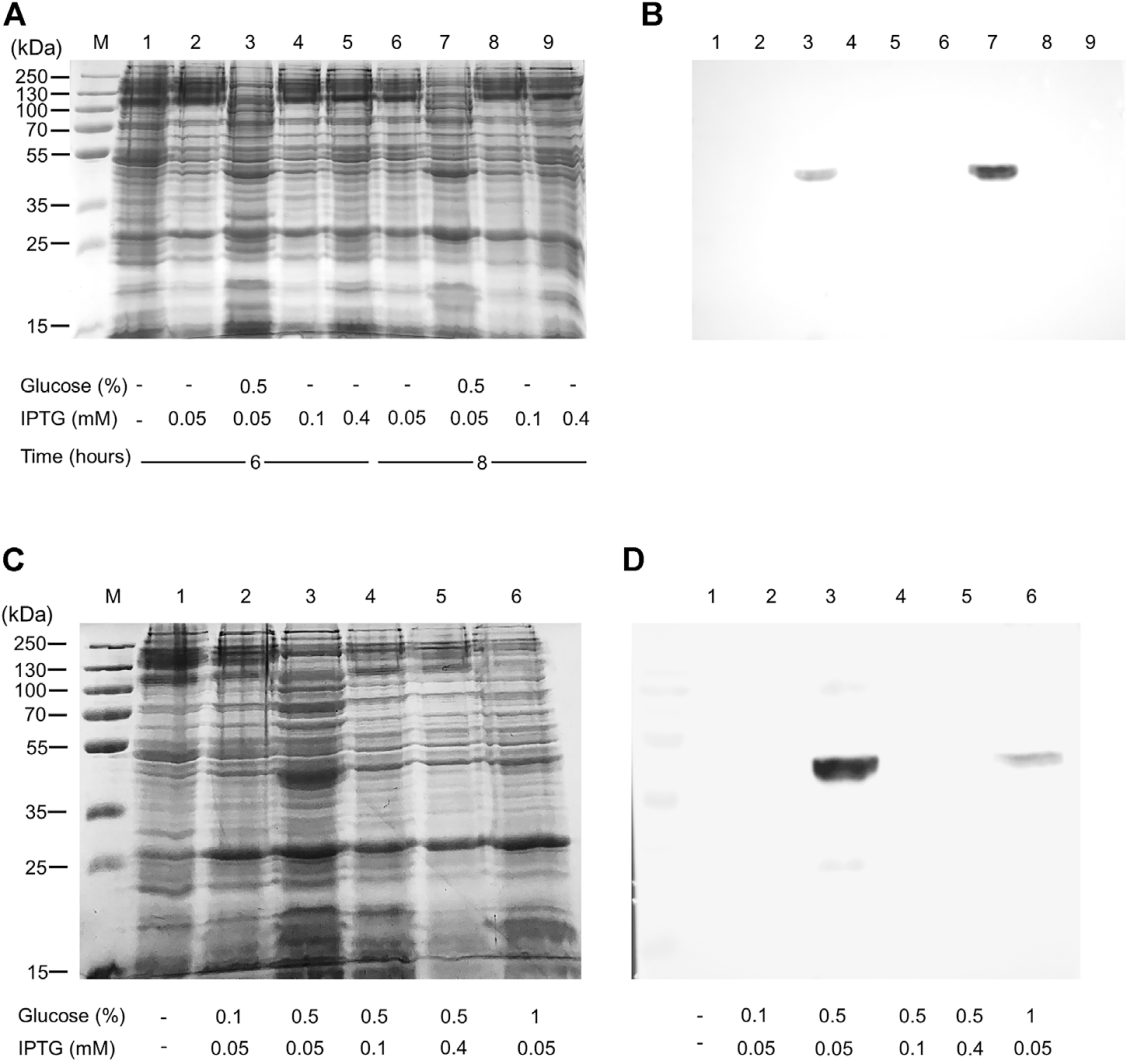
Human xCT expression in *E. coli* BL21. *E. coli* BL21 codon plus was transformed with hxCT-pH6EX3 construct and cultured in the absence or presence of 0.5% glucose. The effect of different IPTG concentrations at different times of induction was also tested. **(A)** SDS-PAGE of insoluble fractions from: 1, uninduced cell lysate; 2–9 induced cell lysates in different conditions: IPTG, glucose concentration and times of growth are indicated in the figure; M, page ruler prestained plus marker; **(B)** Western blotting using an anti-His antibody of the samples loaded as in **(A)**. **(C)** SDS-PAGE of insoluble fractions after 8 h of growth from: 1, uninduced cell lysate; 2–6 induced cell lysates in different conditions: IPTG and glucose concentrations are indicated in the figure; M: page ruler prestained plus marker. **(D)** Western blotting using anti His antibody of the samples loaded as in **(C)**.

**FIGURE 2:**
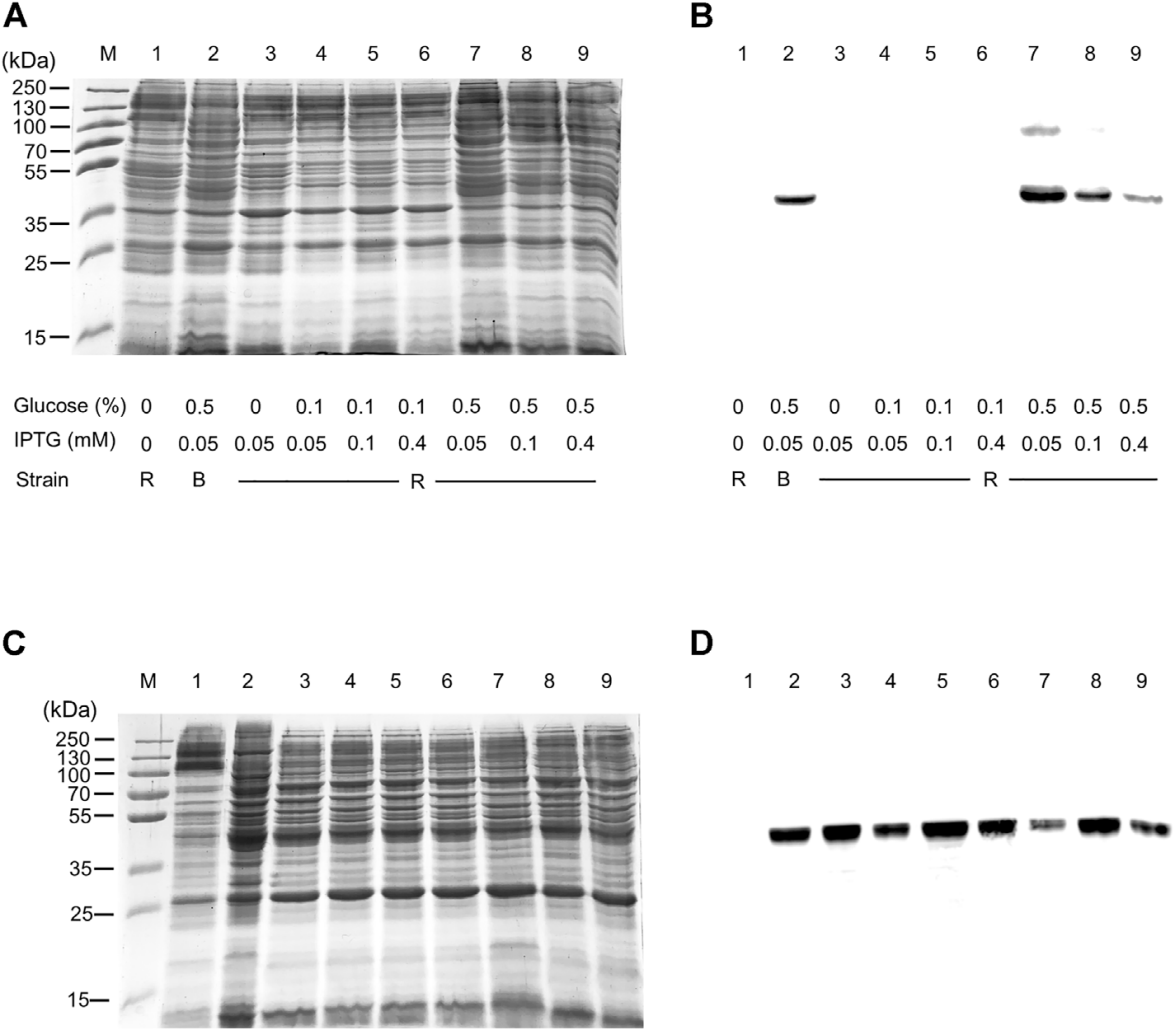
hxCT expression: the effect of strain, medium, and oxygen. *E. coli* BL21 codon plus or Rosetta (DE3) strain was transformed with hxCT-pH6EX3 construct and cultured in the absence or presence of different glucose and IPTG concentrations. **(A)** SDS-PAGE of insoluble fractions after 8 h of growth from: 1, uninduced cell lysate; 2–9 induced cell lysates in different conditions: IPTG and glucose concentration are indicated in the figure; M, page ruler prestained plus marker; R, Rosetta (DE3); B, BL21 codon plus. **(B)** Western blotting using anti His antibody of the samples loaded as in **(A)**. **(C)** SDS-PAGE of insoluble fractions of Rosetta cells after 8 h of growth from: 1, uninduced lysate; 2–9, in presence of 0.5% glucose and 0.05 mM IPTG. In particular, lanes 2 and 3, from 25 ml of LB and Terrific broth, respectively, cultured in a 250 ml Erlenmeyer open flask; lane 4: sample treated as in lane 3 but in presence of plug; lanes 5–7, from 100, 200, and 300 ml of Terrific broth, respectively, cultured in a 1 L Erlenmeyer open flask; lanes 8 and 9, from 200 ml of Terrific broth cultured in a 2 L open and closed Erlenmeyer flask, respectively. **(D)** Western blotting using anti His antibody of the samples loaded as in **(C)**.

**FIGURE 3:**
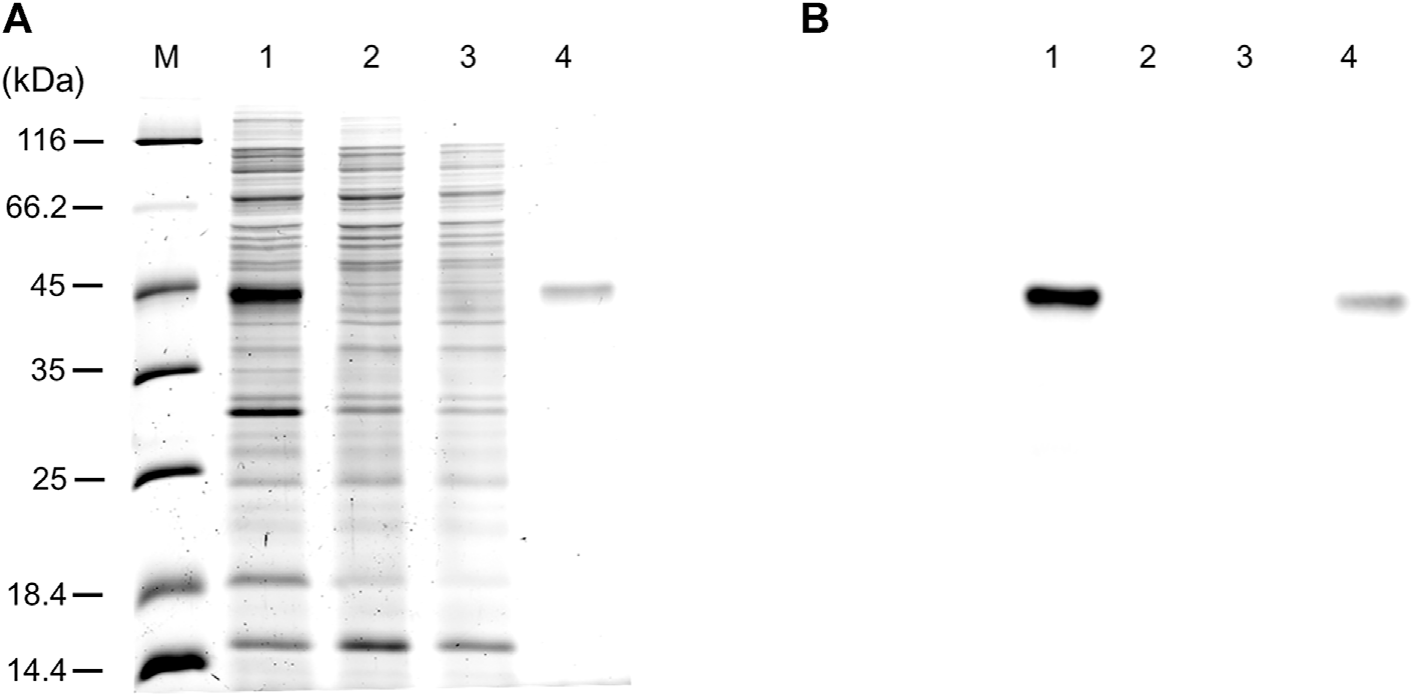
hxCT purification. **(A)** Proteins were separated by SDS–PAGE and visualized by a stain-free procedure as described in materials and methods: lane M, page ruler unstained marker; lane 1: sample as in Figure 2 lane 8 solubilized before IMAC loading; lane 2: flowthrough fraction containing the unbound proteins; lane 3: fraction of the protein eluted with washing buffer added with 10 mM imidazole; lane 4: fraction of the protein eluted with washing buffer added with 500 mM imidazole. **(B)** Western blotting using anti His antibody of the samples loaded as in **(A)**.

The WT human xCT-pH6EX3 construct was firstly used for transforming *E. coli* BL21 codon plus strain adopted to overcome codon bias. A single colony was inoculated and cultured overnight in an appropriate volume of LB broth. After 12–16 h, a 1:20 dilution in fresh LB was performed and the growth was monitored. During the exponential phase of growth (OD ∼ 0.8), the cultures were differently treated as described in the materials and methods section. The IPTG concentration was modulated in a range from 0.05 to 0.4 mM in the presence or absence of 0.5% glucose (Figure 1). Glucose was tested since its addition revealed crucial for the expression of the SLC6A19 amino acid transporter (Galluccio et al., 2020). Indeed, glucose reduces leaky expression by inhibiting lac permease (Winkler and Wilson, 1967) and triggers catabolite repression limiting the transcriptional activation of the RNA polymerase mediated by cAMP (Galluccio et al., 2022a). Following IPTG addition, cells were collected at 6 h or 8 h (Figure 1A, lanes 3 and 7). A protein band with an apparent molecular mass of about 45 kDa was observed in the induced cell lysates only at 0.05 mM IPTG in the presence of 0.5% glucose either at 6 and 8 h. The WB analysis of the same samples confirmed the presence of xCT at the apparent molecular mass of about 45 kDa under the conditions of 0.05 mM IPTG and 0.5% glucose with a much greater amount at 8 h than at 6 h (Figure 1B). Using a low IPTG concentration for improving expression was based on previous works on other human SLC transporters for which lowering IPTG concentration resulted in a slower rate of protein production decreasing the toxicity of the target protein (Förster-Fromme et al., 2017; Galluccio et al., 2020; Galluccio et al., 2022b). The molecular mass observed on the gel is lower than the theoretical mass of the protein, i.e., 57.772 Da, as in the case of other hydrophobic membrane transporters (Galluccio et al., 2009; Rath et al., 2009). The finding that the protein is produced only in the presence of glucose indicates that the catabolite repression phenomenon was crucial for obtaining protein expression (Wanner et al., 1978; Galluccio et al., 2022a). Then, to further investigate the effect of glucose, its influence on protein expression was tested in cells induced with 0.1 and 0.4 mM IPTG (Figure 1C, lanes 4 and 5, respectively). In these conditions, the 45 kDa protein was not produced. The WB analysis of Figure 1D confirmed that xCT was only present at 0.05 mM (Figure 1C lane 3) but not at higher IPTG concentration. On the other way around, decreasing or increasing glucose concentration (Figure 1C lanes 2 and 6, respectively) had a negative effect on protein production. To verify if the protein production could be further improved, a different *E. coli* strain, i.e., Rosetta (DE3) was transformed with the recombinant construct human xCT-pH6EX3. The Rosetta strain was used because it supplies additional tRNA specific for human codon usage with respect to BL21 codon plus (Galluccio et al., 2022a). Then, the dependence on different glucose concentrations (0.1 % and 0.5%) was tested in the presence of three IPTG concentrations (0.05, 0.1, and 0.4 mM) (Figure 2A). As observed for the BL21 strain, the addition of 0.5% glucose was crucial for obtaining the expression of the protein in Rosetta strain (Figure 2B lanes 7–9). Moreover, at 0.05 mM IPTG concentration the expression was higher with respect to 0.1 and 0.4 mM IPTG (Figure 2B lanes 7, 8, and 9, respectively). When compared to the best condition obtained in BL21 (Figure 1C lane 3 and Figure 2B lane 2), a slightly higher over-expression was observed in Rosetta (Figure 2B lane 7). Interestingly, Rosetta cells grew up reaching an O.D. value that is about double with respect to the BL21 cells; the double cell number implies the recovery of a much higher total protein amount (0.5 mg/L of cell culture). During protein expression, we observed the production of foam as a consequence of the bacterial fermentation indicating that the volume and composition of broth could be critical for protein production. Thus, we further tested the effect of oxygen by changing the ratio Broth Volume/Flask Volume (BV/FV) in Terrific Broth (TB). We found that xCT protein production was drastically increased by the reduction of BV/FV, and positively affected also by the absence of plug (open flasks) (Figure 2C). The use of 25 ml of TB in a 250 ml Erlenmeyer flask (Figure 2C, lane 3) caused an increase in protein production with respect to the same BV/FV in LB broth (Figure 2C lane 2). Moreover, the total cell number at the end of the induction experiment increased four times allowing to increase the yield of purified protein per liter of cell culture up to 2 mg/L of cell culture. As expected, by cultivating 100 ml, 200 ml or 300 ml in a 1 L flask, we observed a corresponding reduction in the protein amount (Figure 2C, lanes 5–7). Moreover, the use of the open flask positively influenced xCT production as confirmed by WB (Figure 2D, lanes 3-4 and 8-9). The pellet deriving from 250 ml of Rosetta cells cultured in TB as described above (Figure 2C lane 8), was treated with 24 ml of resuspension buffer containing 20 mM Hepes/Tris pH 7.5 and 300 mM NaCl. Then, the resuspended pellet was sonified and the xCT protein was purified as described in the materials and methods (Figure 3). The purification procedure was slightly modified from that employed for other membrane transporters (Galluccio et al., 2013; Galluccio et al., 2015), by doubling the volume of solubilizing agents, increasing the time of solubilization and the volume of Ni^2+^-chelating resin employed in the purification. The xCT protein was visualized as a single band following a stain-free SDS-PAGE (Figure 3A) after elution with a buffer containing 500 mM imidazole (Figure 3A, lane 4). The identity of the protein was confirmed by western blotting using an anti-His antibody (Figure 3B lane 4). Noteworthy, the binding of the 6His-xCT protein was effective since no reaction against the anti-His antibody was observed neither in the flow through (Figure 3B, lane 2) nor in the washing fraction containing 10 mM imidazole (Figure 3B, lane 3).

### Functional and kinetic characterization of xCT in proteoliposomes

**FIGURE 4:**
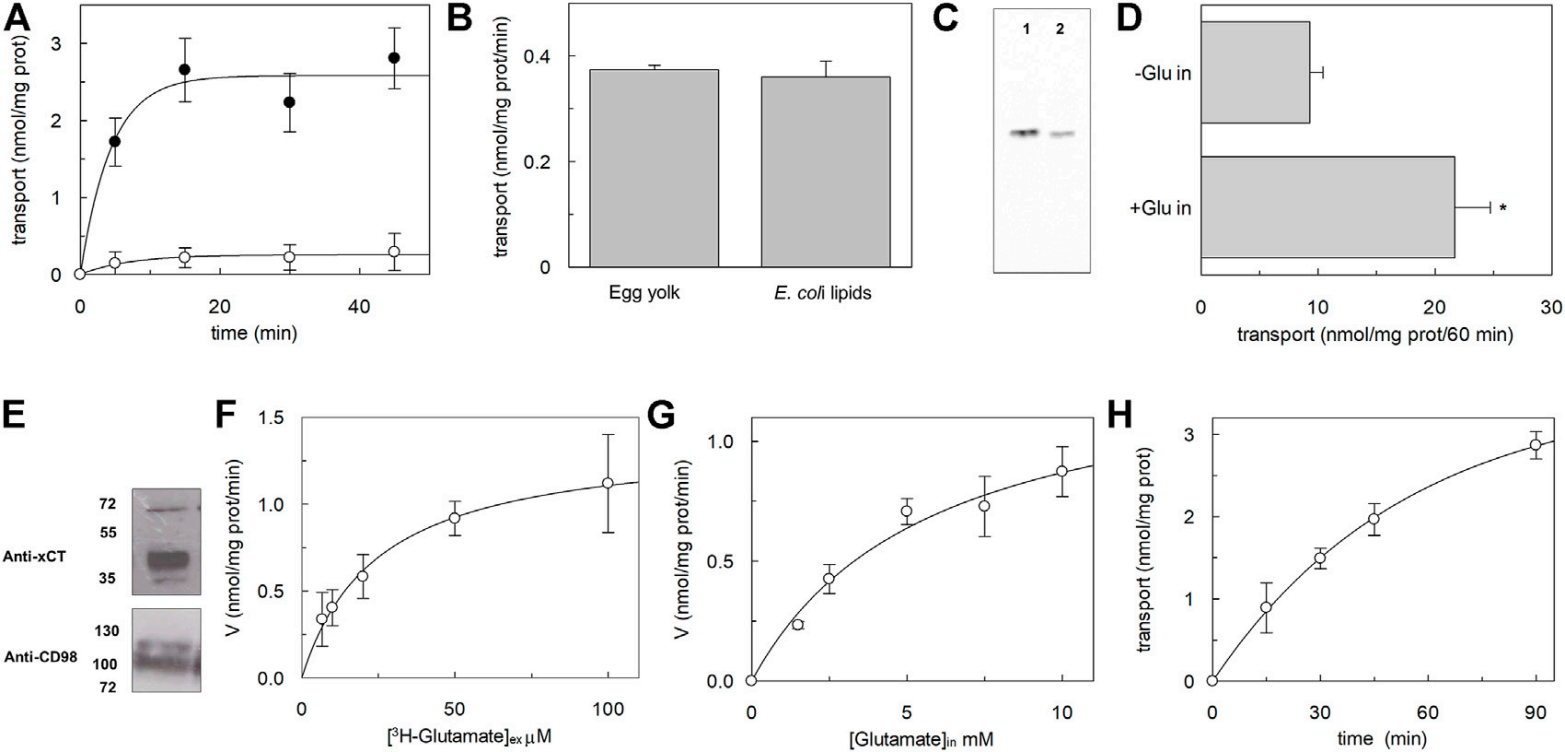
Biochemical characterization of hxCT reconstituted in proteoliposomes. In **(A)**, time course analysis of reconstituted xCT. Purified protein was reconstituted as described in materials and methods. The transport was started by adding 10 μM [³H]-glutamate to proteoliposomes without (o) or with 10 mM internal glutamate (●), and stopped at indicated times. Data are plotted using first order rate equation. Data are presented as mean ± SD of four independent experiments. In **(B)**, effect of *E. coli* lipids. Purified protein was reconstituted as described in materials and methods. The transport was started by adding 10 μM [³H]-glutamate to proteoliposomes prepared with phosphatidyl choline from egg yolk or with a mixture of phosphatidyl choline from egg yolk and bacterial lipids (70:30). The transport was stopped after 20 min. In **(C)**, Western blot analysis. The purified protein (lane 1) and reconstituted protein (lane 2) were loaded onto a 12% SDS-PAGE for Western blot analysis with anti-His antibody. In **(D)**, transport of native xCT extracted from A549 cells. Cell extracts were prepared and reconstituted as described in materials and methods. The transport was started by adding 10 μM [³H]-glutamate to proteoliposomes without or with 10 mM internal glutamate and stopped after 60 min as indicated in the figure. *Significantly different from the control (-Glu in) as estimated by Student’s *t*-test (*p* < 0.01). In **(E)** Western blot analysis. The A549 pellet was solubilized as described in material and methods and 50 μg were loaded onto a 12% SDS-PAGE for Western blot analysis with indicated antibodies. In **(F,G)** kinetic analysis of the recombinant xCT reconstituted in proteoliposomes as described in materials and methods. In **(F)**, the transport was measured in 10 s by adding indicated concentrations of [³H]-glutamate to proteoliposomes containing 10 mM internal glutamate, for evaluation of external Km for glutamate. In **(G)**, the transport was measured in 10 s by adding 10 μM [³H]-glutamate to proteoliposomes containing indicated concentrations of internal glutamate, for evaluation of internal Km for glutamate. Data are plotted using the Michaelis-Menten equation. Data are presented as mean ± SD of three independent experiments and transport rate is measured as nmol/mg prot/min (V). In **(H)**, time course analysis of reconstituted xCT. Purified protein was reconstituted as described in materials and methods. The transport was started by adding 10 μM [³H]-glutamate to proteoliposomes containing 5 mM internal cystine and stopped at indicated times. Data are plotted using first-order rate equation. Data are presented as mean ± SD of three independent experiments.

The major scope of this work was obtaining a recombinant xCT at a relatively low cost available for further functional and kinetic characterization, to shed light on still unknown features of xCT biology. Once obtained the purified protein, we looked at its functional state by performing a transport assay in proteoliposomes using a previously described procedure (Cosco et al., 2020), with some adaptations. Starting from the few known functional properties of xCT (Sato et al., 1999), the transport activity was measured as the exchange of external [^3^H]-glutamate with internal glutamate (Figure 4A). The homoexchange reaction was used since this procedure allows obtaining a higher accumulation of the radioisotope inside the vesicles with respect to the heteroexchange procedure (Scalise et al., 2020) facilitating the activity measurements. The time course analysis showed an accumulation of radiolabelled glutamate with an initial transport rate of 0.59 ± 0.1 nmol * mg prot^−1^ * min^−1^, reaching the radioisotope equilibration at 30 min. As a control, in the same experiments, liposomes without incorporated proteins were used and no accumulation of radiolabelled substrate was observed (Figure 4A). The obligatory antiport mechanism was also evaluated by measuring the uptake of [^3^H]-glutamate in the absence of internal glutamate; as it is shown in Figure 4A, virtually no accumulation of [^3^H]-glutamate was observed in the absence of a counter-substrate. As in the case of LAT1, the reconstitution of xCT was performed with a protein/lipid ratio of 1:2,000. Considering that xCT is over-expressed in *E. coli* cells, the effect of bacterial lipids on the transport activity of xCT was evaluated; as it is shown in Figure 4B, the [^3^H]-glutamate uptake was virtually not affected by the presence of bacterial lipids. It has to be highlighted that the amount of protein inserted into the proteoliposome membrane accounted for roughly 30% of the total, as evaluated by Western blot analysis reported in Figure 4C, moving from a previously adopted methodology; this fraction represents the protein, available for functional studies, most probably corresponding to that correctly folded protein and is in the range of the fraction of other functional transporters incorporated in proteoliposomes [(Galluccio et al., 2022b) and refs herein]. To compare the transport features of recombinant xCT to that of the native one, the protein was solubilized from A549 cell pellets and reconstituted in proteoliposomes (Figure 4D). It has to be stressed that in this condition, xCT was associated with the ancillary protein CD98, being extracted from intact cells, as shown by Western blot analysis (Figure 4E). The [^3^H]-glutamate uptake was stimulated by the presence of internal glutamate, being in good agreement with the antiport mode and with the data obtained using the recombinant xCT (Figure 4A). These results confirmed that the recombinant protein is in a functionally active state and that does not require the heavy chain CD98 for catalysing amino acid transport, as previously demonstrated for the fifth member of SLC7 family, LAT1 (Napolitano et al., 2015). Indeed, it is plausible that also in the case of xCT, the ancillary protein CD98 is required in cells for routing the light chain to the definitive location in the plasma membrane, with no effects on the intrinsic transport activity of the heterodimer. The [^3^H]-glutamate uptake measured in the absence of internal glutamate (Figure 4B), which is higher than that measured with the recombinant protein, can be ascribed to some interferences by other glutamate specific uniport transporters present in the total cell extract. To deepen the knowledge of the xCT, kinetic analysis was performed using the recombinant protein reconstituted in proteoliposomes (Figures 4F,G); the derived apparent external Km for glutamate was 25 ± 7.8 μM (Figure 4F), whereas the apparent internal Km for glutamate was 5.8 ± 1.6 mM (Figure 4G). It has to be stressed that apparent Km values are reported since a bi-substrate kinetic analysis for defining the transport mechanism and, hence the substrate independent actual Km values, still needs to be described and will be dealt with in next studies. Interestingly, the external Km for glutamate was already calculated in the very early study (Sato et al., 1999), being similar to that measured in this work, confirming that the properties of the recombinant protein overlap those of the native protein. As above-mentioned, due to difficulties in controlling the composition of cytosolic space in an intact cell system, the internal Km was not known; we here show a profoundly different value from the external one. This finding is in good agreement with a side-right out orientation of the transporter in liposomes that resulted in a kinetic asymmetry, as in the case of LAT1 (Napolitano et al., 2015). Finally, to ascertain the actual occurrence of a glutamate/cystine exchange, the uptake of radiolabelled glutamate was measured in exchange with internal cystine (Figure 4H). Again in this case, a measurable transport was observed with an initial transport rate of 0.1 nmol * mg prot^−1^ * min^−1^ ± 0.05 being, as expected, lower than the homoexchange.

## Conclusion

The availability of recombinant human xCT in an active form was mandatory for studying the function of this transporter. Even though bacteria represent the easiest and cheapest tool for obtaining recombinant proteins, expression of membrane human proteins is often very difficult in this host (Galluccio et al., 2022a). Therefore, strategies based on metabolic and growth condition changes have been adopted for xCT, starting from previous knowledge of other SLC bacterial expression. In the case of xCT, the novel finding which revealed crucial for protein production was the BV/FV ratio, which indirectly conditions the oxygen partial pressure. The abundantly over-expressed protein was then tested for its suitability for physiological activity assays. Indeed, the recombinant xCT was able to catalyse in proteoliposomes, the most common transport reactions previously tested in intact cells, thus confirming the active state of the protein. A comparison with the activity of the protein extracted from a cell line containing CD98, correlated well with the assumption that the xCT moiety was responsible for the amino acid antiport reaction. The identification of the optimal growth conditions and the reconstitution of xCT in proteoliposomes open the way to further investigations devoted to describing still unknown physiological features of this important transporter, with outcomes in human health and pharmacology.

## Data Availability

The raw data supporting the conclusion of this article will be made available by the authors, without undue reservation.

## References

[B1] Chio I. I. C. Tuveson D. A. (2017). ROS in cancer: The burning question. Trends Mol. Med. 23 (5), 411–429. 10.1016/j.molmed.2017.03.004 28427863 PMC5462452

[B2] Combs J. A. DeNicola G. M. (2019). The non-essential amino acid cysteine becomes essential for tumor proliferation and survival. Cancers (Basel) 11 (5), E678. 10.3390/cancers11050678 31100816 PMC6562400

[B3] Cosco J. Scalise M. Colas C. Galluccio M. Martini R. Rovella F. (2020). ATP modulates SLC7A5 (LAT1) synergistically with cholesterol. Sci. Rep. 10 (1), 16738. 10.1038/s41598-020-73757-y 33028978 PMC7541457

[B4] Dulley J. R. Grieve P. A. (1975). A simple technique for eliminating interference by detergents in the Lowry method of protein determination. Anal. Biochem. 64 (1), 136–141. 10.1016/0003-2697(75)90415-7 1137083

[B5] Förster-Fromme K. Schneider S. Sprenger G. A. Albermann C. (2017). Functional expression of a human GDP-L-fucose transporter in *Escherichia coli* . Biotechnol. Lett. 39 (2), 219–226. 10.1007/s10529-016-2233-x 27738779

[B6] Galluccio M. Console L. Pochini L. Scalise M. Giangregorio N. Indiveri C. (2022). Strategies for successful over-expression of human membrane transport systems using bacterial hosts: Future perspectives. Int. J. Mol. Sci. 23 (7), 3823. 10.3390/ijms23073823 35409183 PMC8998559

[B7] Galluccio M. Mazza T. Scalise M. Sarubbi M. C. Indiveri C. (2022). Bacterial over-expression of functionally active human CT2 (SLC22A16) carnitine transporter. Mol. Biol. Rep. 49, 8185–8193. 10.1007/s11033-022-07491-1 35608746

[B8] Galluccio M. Pantanella M. Giudice D. Brescia S. Indiveri C. (2020). Low temperature bacterial expression of the neutral amino acid transporters SLC1A5 (ASCT2), and SLC6A19 (B0AT1). Mol. Biol. Rep. 47 (9), 7283–7289. 10.1007/s11033-020-05717-8 32772343 PMC7415195

[B9] Galluccio M. Pingitore P. Scalise M. Indiveri C. (2013). Cloning, large scale over-expression in *E. coli* and purification of the components of the human LAT 1 (SLC7A5) amino acid transporter. Protein J. 32 (6), 442–448. 10.1007/s10930-013-9503-4 23912240

[B10] Galluccio M. Pochini L. Amelio L. Accardi R. Tommasino M. Indiveri C. (2009). Over-expression in *E. coli* and purification of the human OCTN1 transport protein. Protein Expr. Purif. 68 (2), 215–220. 10.1016/j.pep.2009.06.015 19567267

[B11] Galluccio M. Pochini L. Peta V. Ianni M. Scalise M. Indiveri C. (2015). Functional and molecular effects of mercury compounds on the human OCTN1 cation transporter: C50 and C136 are the targets for potent inhibition. Toxicol. Sci. 144 (1), 105–113. 10.1093/toxsci/kfu259 25490951

[B12] Ghasemitarei M. Yusupov M. Razzokov J. Shokri B. Bogaerts A. (2019). Transport of cystine across xC(-) antiporter. Arch. Biochem. Biophys. 664, 117–126. 10.1016/j.abb.2019.01.039 30738038

[B13] Go Y. M. Jones D. P. (2008). Redox compartmentalization in eukaryotic cells. Biochim. Biophys. Acta 1780 (11), 1273–1290. 10.1016/j.bbagen.2008.01.011 18267127 PMC2601570

[B14] Ji X. Qian J. Rahman S. M. J. Siska P. J. Zou Y. Harris B. K. (2018). xCT (SLC7A11)-mediated metabolic reprogramming promotes non-small cell lung cancer progression. Oncogene 37 (36), 5007–5019. 10.1038/s41388-018-0307-z 29789716 PMC6127081

[B15] Jong C. J. Azuma J. Schaffer S. (2012). Mechanism underlying the antioxidant activity of taurine: Prevention of mitochondrial oxidant production. Amino Acids 42 (6), 2223–2232. 10.1007/s00726-011-0962-7 21691752

[B16] Jyotsana N. Ta K. T. DelGiorno K. E. (2022). The role of cystine/glutamate antiporter slc7a11/xCT in the pathophysiology of cancer. Front. Oncol. 12, 858462. 10.3389/fonc.2022.858462 35280777 PMC8904967

[B17] Kinoshita H. Okabe H. Beppu T. Chikamoto A. Hayashi H. Imai K. (2013). Cystine/glutamic acid transporter is a novel marker for predicting poor survival in patients with hepatocellular carcinoma. Oncol. Rep. 29 (2), 685–689. 10.3892/or.2012.2162 23229496

[B18] Koppula P. Zhang Y. Zhuang L. Gan B. (2018). Amino acid transporter SLC7A11/xCT at the crossroads of regulating redox homeostasis and nutrient dependency of cancer. Cancer Commun. 38 (1), 12. 10.1186/s40880-018-0288-x PMC5993148 29764521

[B19] Koppula P. Zhuang L. Gan B. (2021). Cystine transporter slc7a11/xCT in cancer: ferroptosis, nutrient dependency, and cancer therapy. Protein Cell 12 (8), 599–620. 10.1007/s13238-020-00789-5 33000412 PMC8310547

[B20] Laemmli U. K. (1970). Cleavage of structural proteins during the assembly of the head of bacteriophage T4. Nature 227 (5259), 680–685. 10.1038/227680a0 5432063

[B21] Lin W. Wang C. Liu G. Bi C. Wang X. Zhou Q. (2020). SLC7A11/xCT in cancer: biological functions and therapeutic implications. Am. J. Cancer Res. 10 (10), 3106–3126. 33163260 PMC7642655

[B22] Liu J. Xia X. Huang P. (2020). xCT: A critical molecule that links cancer metabolism to redox signaling. Mol. Ther. 28 (11), 2358–2366. 10.1016/j.ymthe.2020.08.021 32931751 PMC7647670

[B23] Lu S. C. (2009). Regulation of glutathione synthesis. Mol. Asp. Med. 30 (1-2), 42–59. 10.1016/j.mam.2008.05.005 PMC2704241 18601945

[B24] Mesci P. Zaidi S. Lobsiger C. S. Millecamps S. Escartin C. Seilhean D. (2015). System xC- is a mediator of microglial function and its deletion slows symptoms in amyotrophic lateral sclerosis mice. Brain 138 (1), 53–68. 10.1093/brain/awu312 25384799 PMC4441079

[B25] Napolitano L. Scalise M. Galluccio M. Pochini L. Albanese L. M. Indiveri C. (2015). LAT1 is the transport competent unit of the LAT1/CD98 heterodimeric amino acid transporter. Int. J. Biochem. Cell Biol. 67, 25–33. 10.1016/j.biocel.2015.08.004 26256001

[B26] Oda K. Lee Y. Wiriyasermkul P. Tanaka Y. Takemoto M. Yamashita K. (2020). Consensus mutagenesis approach improves the thermal stability of system xc (-) transporter, xCT, and enables cryo-EM analyses. Protein Sci. 29 (12), 2398–2407. 10.1002/pro.3966 33016372 PMC7679960

[B27] Pampliega O. Domercq M. Soria F. N. Villoslada P. Rodriguez-Antiguedad A. Matute C. (2011). Increased expression of cystine/glutamate antiporter in multiple sclerosis. J. Neuroinflammation 8, 63. 10.1186/1742-2094-8-63 21639880 PMC3117706

[B28] Parker J. L. Deme J. C. Kolokouris D. Kuteyi G. Biggin P. C. Lea S. M. (2021). Molecular basis for redox control by the human cystine/glutamate antiporter system xc<sup/>. Nat. Commun. 12 (1), 7147. 10.1038/s41467-021-27414-1 34880232 PMC8654953

[B29] Rath A. Glibowicka M. Nadeau V. G. Chen G. Deber C. M. (2009). Detergent binding explains anomalous SDS-PAGE migration of membrane proteins. Proc. Natl. Acad. Sci. U. S. A. 106 (6), 1760–1765. 10.1073/pnas.0813167106 19181854 PMC2644111

[B30] Sato H. TaMbaM. Ishii T. Bannai S. (1999). Cloning and expression of a plasma membrane cystine/glutamate exchange transporter composed of two distinct proteins. J. Biol. Chem. 274 (17), 11455–11458. 10.1074/jbc.274.17.11455 10206947

[B31] Sato M. Kusumi R. Hamashima S. Kobayashi S. Sasaki S. Komiyama Y. (2018). The ferroptosis inducer erastin irreversibly inhibits system x(c)- and synergizes with cisplatin to increase cisplatin's cytotoxicity in cancer cells. Sci. Rep. 8, 968. 10.1038/s41598-018-19213-4 29343855 PMC5772355

[B32] Scalise M. Mazza T. Pappacoda G. Pochini L. Cosco J. Rovella F. (2020). The human SLC1A5 neutral amino acid transporter catalyzes a pH-dependent glutamate/glutamine antiport, as well. Front. Cell Dev. Biol. 8, 603. 10.3389/fcell.2020.00603 32733894 PMC7360689

[B33] Shin S. S. Jeong B. S. Wall B. A. Li J. Shan N. L. Wen Y. (2018). Participation of xCT in melanoma cell proliferation *in vitro* and tumorigenesis *in vivo* . Oncogenesis 7 (11), 86. 10.1038/s41389-018-0098-7 30425240 PMC6234219

[B34] Stipanuk M. H. Dominy J. E. Lee J. I. Coloso R. M. (2006). Mammalian cysteine metabolism: New insights into regulation of cysteine metabolism. J. Nutr. 136 (6), 1652S–1659s. 10.1093/jn/136.6.1652S 16702335

[B35] Wanner B. L. Kodaira R. Neidhardt F. C. (1978). Regulation of lac operon expression: reappraisal of the theory of catabolite repression. J. Bacteriol. 136 (3), 947–954. 10.1128/JB.136.3.947-954.1978 214424 PMC218529

[B36] Winkler H. H. Wilson T. H. (1967). Inhibition of beta-galactoside transport by substrates of the glucose transport system in *Escherichia coli* . Biochim. Biophys. Acta 135 (5), 1030–1051. 10.1016/0005-2736(67)90073-9 4863902

[B37] Yan R. Xie E. Li Y. Li J. Zhang Y. Chi X. (2022). The structure of erastin-bound xCT-4F2hc complex reveals molecular mechanisms underlying erastin-induced ferroptosis. Cell Res. 32, 687–690. 10.1038/s41422-022-00642-w 35352032 PMC9253326

